# Immunoglobulin G Expression in Human Sperm and Possible Functional Significance

**DOI:** 10.1038/srep20166

**Published:** 2016-02-01

**Authors:** Meiling Yan, Xiaoyu Zhang, Qinxue Pu, Tao Huang, Qingdong Xie, Yan Wang, Jing Li, Yun Wang, Huan Gu, Tianhua Huang, Zhiling Li, Jiang Gu

**Affiliations:** 1 Guangdong Provincial Key Laboratory of Infectious Diseases and Molecular Immunopathology, Department of Pathology and Pathophysiology, Shantou University Medical College, Shantou, Guangdong, China; 2 Research Center for Reproductive Medicine, Shantou University Medical College, Shantou, China; 3 Neuroscience Center, Shantou University Medical College, Shantou, China; 4 Jinxin Research Institute for Reproductive Medicine and Genetics, Chengdu Jinjiang Hospital for Maternal and Child Health Care, Chengdu, Sichuan, China; 5 Reproductive Center, The First Affiliated Hospital of Shantou University Medical College, Shantou University, Shantou, Guangdong, China

## Abstract

Immunoglobulin G (IgG), the major molecule of the immune system, which was traditionally thought to be produced by differentiated B-lymphocytes, had recently been found in non-immune cells including spermatozoa of rabbit testis. To study if human sperms could produce IgG that might play a role in fertilization, we employed immunofluorescent staining, Western blot, *in situ* hybridization, RT-PCR (reverse transcription polymerase chain reaction) and immunoelectron microscope and found that human sperms were capable of synthesizing IgG. IgG protein and mRNA were detected in the cytoplasm, mainly the neck region of the sperm and IgG immunoreactivity was found to cover the entire sperm cell. The essential enzymes necessary for IgG synthesis and class switching, RAG1 (recombination activating gene 1), RAG2 (recombination activating gene 2) and AID (activation-induced cytidine deaminase), were also detected in the sperm cells. Furthermore, we found that anti-IgG antibody could inhibit sperm from penetrating Zona-free hamster egg with statistical significance. These discoveries suggested that immunoglobulin G could be produced by human sperms and it might play a role during fertilization.

Recent discovery of IgG expression in non-lymphocytes has challenged the classical concept that this important immune molecule can only be synthesized and released by mature B lymphocytes and plasma cells. It is now known that cell types that can synthesize IgG include cancer cells and normal cells such as hepatocytes, placental trophoblasts, neurons, cells in the eye and the testis^1^,^2^,^3^,^4^,^5^,^6^,^7^,^8^. The presence of IgG and IgA in mammalian sperm was reported in a few studies including the detection of immunoglobulin related transcripts in a single human sperm^9^,^10^,^11^,^12^,^13^. IgG was also abundantly detected in fluid from the distal end of the ductus epididymidis, accessory glands and spermatozo cells in rabbits^14^,^15^. Previous studies based on the results of RT-PCR seemed to suggest that neonatal Fc receptor (FcRn) was shown to express in the reproductive system, where IgG can be transported across the reproductive epithelium via such receptors^16^. These findings suggested that IgG might be expressed by human sperm, but direct evidence to support this assumption has not been forthcoming.

The functions of IgG produced by non-lymphocytes have been investigated. Tumor-derived IgG is thought to promote the growth and survival of cancer cells^7^. In brain, IgG might act as a self-protective factor via a mechanism of enhancing microglial endocytosis and release of tumor necrosis factor-α (TNF-α) or by neutralizing complement factors^17^,^18^.

Margni *et al.* isolated a special type of IgG from pregnancy women’s sera^19^. These IgG molecules had an aberrant high mannose-type oligosaccharide residue in only one of its Fab fragments and were named asymmetric IgG^20^,^21^. The oligosaccharide residue in Fab fragment could bind to concanavalin A with which the asymmetric IgG can be separated from the symmetric IgG that had no oligosaccharide on its Fab^20^,^22^. Further studies suggested that asymmetrically glycosylated IgG failed to trigger immune effector mechanisms, and played a protective role in fetal survival^19^,^21^,^23^. Similar phenomenon was observed in ovarian and endometrial cancer patients by another group^24^. These studies indicated that non-lymphocyte produced IgG especially asymmetric IgG may play an important role in tumor and fetal evasion of the host immune system.

Until now, there are little information about sperm IgG expression in humans and its possible functions. In this study, biochemical, immunological and molecular biological experiments were performed to study the gene expression and localization of IgG in human spermatozoa. In addition, we purified human sperm IgG and performed indirect ELISA to exam if human sperm could synthesize asymmetric IgG. Finally, the possible roles of IgG in human sperm fertilization were investigated with a zona-free hamster egg penetration assay. We found that human sperms could express IgG which might play a role in fertilization.

## Results

### IgG molecule was detected in human sperm

**Figure 1:**
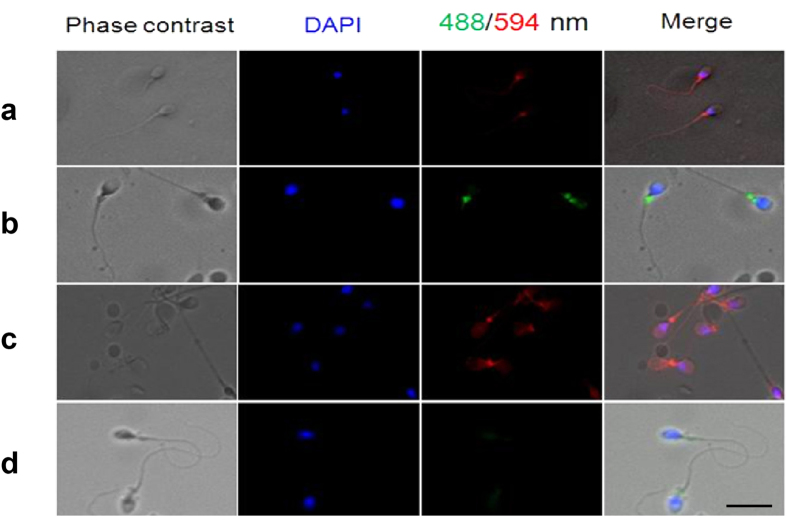
Immunofluorescence stains show that human sperms were positive for IgG. (**a**) The primary antibody is rabbit anti-human IgG γ chain, the secondary antibody is donkey anti-rabbit IgG (H + L) labeled with Alexa Fluor® 594 and the positive stain is red in color. (**b**) The primary antibody is mouse anti-human IgG κ chain antibody, the secondary antibody is goat anti-mouse labeled with Alexa Fluor® 488 and the positive stain is green in color. (**c**) The primary antibody is rabbit anti-human IgG λ chain, and the secondary antibody is donkey anti-rabbit Alexa Fluor® 594 IgG (H + L) and the positive stain is red in color. (**d**) Negative control. DAPI was used to stain the nucleus of sperm (blue). The scale bar is 30 μm for all photos.

**Figure 2:**
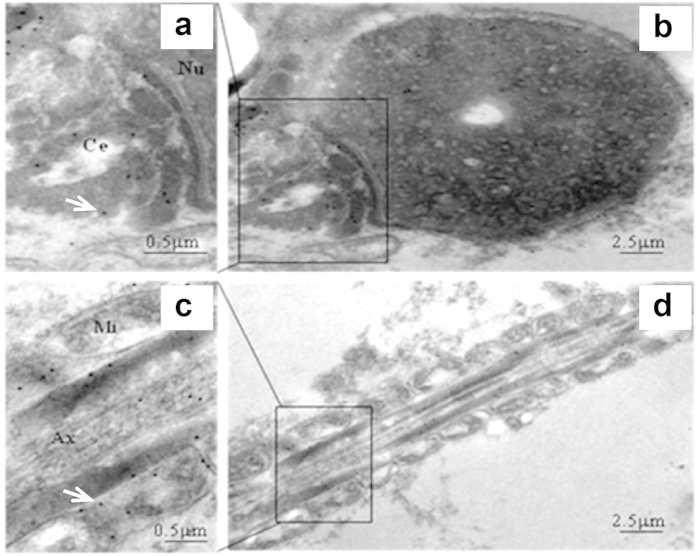
IgG could be detected in the neck and the tail of human sperm with immuno-electron microscopy. Normal human sperm was processed and stained with rabbit anti-human IgG antibody (γ chain specific) and the secondary antibody to rabbit IgG was labeled with 10 nm colloidal gold. The positive stain was shown by highly electron-dense gold particles. (**a**) The neck region of sperm contains abundant gold particles (white arrow) showing the subcellular distribution of IgG. Nu is nucleus and Ce is centriole. (**b**) Lower magnification of (**a**). (**c**) The middle piece of a sperm tail. The gold particles (white arrow) depict IgG distribution. Mi is mitochondria and Ax is axoneme. (**d**) Lower magnification of (**c**). The results demonstrate that both the neck region and the middle piece of the tail of human sperm contain synthesis IgG γ chain molecule.

Immunofluorescence staining (IF) was used to localize the distribution of IgG in human spermatozoa with FITC-labeled specific antibodies to human IgG γ, κ, and λ chain. The results showed that IgG immunoreactivity was mainly located in the neck region of human spermatozoa (Fig. 1). The IgG locations in human spermatozoa were further confirmed with immuno-gold electron microscopy (IEM) (Fig. 2a,b). The locations of IgG demonstrated with immune electronic microscope (IEM) were consistent with that seen in IF staining. However, we also detected IgG expression in the middle piece of sperm tail with the IEM method (Fig. 2c,d).

**Figure 3:**
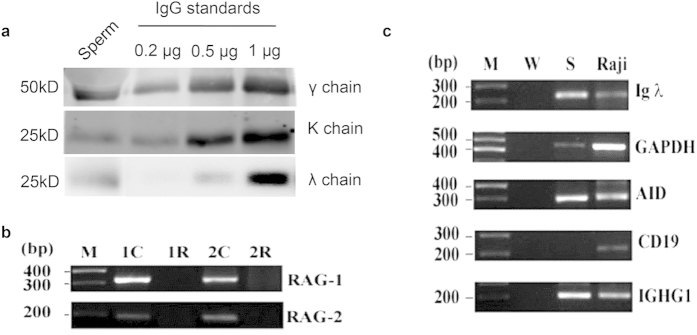
Western blot assay indicated the expression of IgG. RT-PCR amplification detected the expression of IgG mRNA in sperm. (**a**) Results of Western blot. The first lane is total protein extracted from human sperm (56 μg), the second lane to the forth lane is 0.2 μg /0.5 μg /2 μg human standard IgG molecule respectively (positive control). Anti-human IgG γ chain antibody, anti-IgG λ chain antibody and anti-IgG κ chain antibody were used as primary antibodies respectively. Molecular weight of protein was labeled on the right of each panel. (**b**) Expression of RAG-1 (327 bp) and RAG-2 (193 bp) genes were detected in human spermatozoa. Lane 1 is PCR product of RAG1 and RAG2 gene extracted from Raji cells (positive control). Lane 2 is DNase treated cDNA of Raji cells was used as a template (negative control). Lane 3 is cDNA of normal human sperm extract was used as a template. RAG1 and RAG2 genes were amplified. Lane 4 is DNase treated template of normal human sperm. No RAG1 or RAG2 gene was detected. (**c**) RT-PCR amplification of mRNA transcripts of IgG λ chain, AID, IGHG1, CD19 and GAPDH in human sperm. There was no contamination from B cells in human sperm since CD19 was negative. Human sperm could express transcripts of IgG λ chain, AID, IGHG1 and GAPDH. M is DNA ladder, W is water as a negative control, S represents human sperm, Raji cell was used as the positive control. CD19 is a marker for Raji cell. The results show that human sperm can express the essential genes for immunoglobulin G V-D-J rearrangement. In addition, human sperm can synthesis IgG in both the protein level and the mRNA levels.

Western blot detected protein bands of IgG λ/κ chain and IgG γ chain in human sperm protein extraction with molecular size of 25 kD and 50 kD respectively (Fig. 3a). Different IgG-related antibodies were shown to detect the same molecular weights of proteins from both human IgG standard and human sperm extraction.

### IgG mRNA and the essential enzymes for IgG synthesis were detected in human sperm

Expressions of the IgG synthesizing enzymes RAG1 and RAG2 genes in human sperm were detected with RT-PCR (Fig. 3b). Subsequently, IgG λ chain, AID, and IGHG1 were detected in human sperm as shown in Fig. 3c. The absence of CD19 expression in human sperm ruled out the possible contaminations by B lymphocytes during sperm preparations.

**Figure 4:**
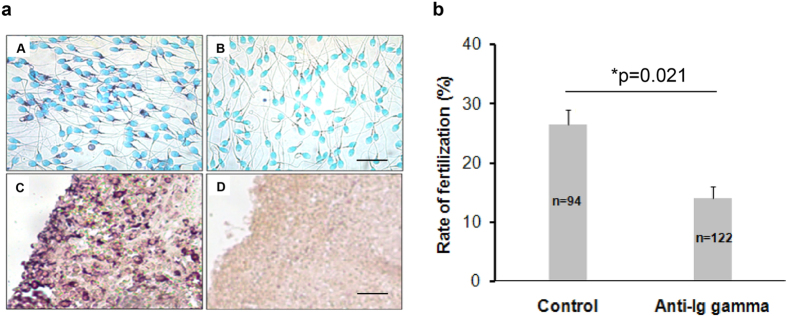
(**a**) *In situ* hybridization assay detected the expression and location of IgG mRNA. (A,B) were human sperms incubated with anti-sense probe and sense probe against IgG γ chain respectively. The positive stain (purple) in (A) demonstrated mRNA expression of IgG which was mainly located in the neck of human sperm. There was no positive stain with sense probe as shown in (B). (C,D) were human normal spleen tissue served as additional controls. (C) was incubated with anti-sense probe against IgG γ chain (positive control) and (D) was incubated with sense probe against IgG γ chain (negative control). Bar was 20 μm in (A,B). Bar was 25 μm in (C,D). (**b**) Anti-IgG antibody could inhibit sperm-egg penetration. After being pre-incubated with anti-human IgG antibody (γ chain specific) for 4 h at 37 °C, zona-free hamster egg-sperm penetration assay were performed and the penetration ratio was calculated. The sperms without anti-IgG antibody treatment were used as a control for comparison. Data show means ± SE of four independent experiments. The mean penetration ratio in the treatment group is 27%, while the mean ratio in the control group is 15%. The difference was statistically significant between the two groups (*p < 0.05, One way ANOVA, Dunnet’s post-hoc test). n is the number of zona-free hamster eggs used in the penetration assay for each group. The result shows that IgG produced by sperm may play a role in sperm-egg fusion.

*In situ* hybridization (ISH) method was used to detect IgG mRNA expression in human sperm with a probe for IgG1 heavy chain constant region (IGHG1) (Fig. 4aA). As a positive control, IGHG1 was found in normal spleen tissue (Fig. 4aC). When the sense probe of IGHG1 was used, both human sperm (Fig. 4aB) and spleen tissue (Fig. 4aD) gave negative results. The results were presented in Fig. 4a.

### About 23% of IgG extracted from sperm were Fab glycosylated

In order to determine if sperm produced IgG is asymmetric, we used concanavalin A-sepharose column to isolate asymmetric IgG, collecting the washing buffer which was the unbound IgG (symmetric IgG molecule) and other protein mixture. Then we used a human IgG ELISA kit to test the concentration of total IgG and unbound IgG in total sperm protein and the percentage was calculated. We found that human sperm IgG contained about 23% asymmetric IgG.

### Anti-IgG antibody inhibited egg-sperm fertilization

The effect of antibody against human IgG on the fertilization of human sperm was examined with zona-free hamster egg-sperm penetration assay^25^. Rabbit anti-human IgG antibody (γ chain specific) was used in this experiment. Upon incubation of motile sperm with 5.7 μg/ml rabbit anti-IgG antibody, zona-free hamster egg-sperm penetration assay was performed. The rate of fertilization was found to be significantly inhibited in the antibody treated group in comparison to that of the negative control without primary antibody treatment. The difference in sperm penetration rates between the rabbit anti-IgG antibody (γ chain specific) treatment group (n = 122) and the negative control group (n = 94) was shown to be statistically significantly (P = 0.021) (Fig. 4b).

## Discussion

In this study, we demonstrated that IgG was expressed by sperm cells. This was detected not only with immunohistochemistry to detect IgG antigens but also with *in situ* hybridization and RT-PCR to detect its mRNA. In addition, the essential enzymes for IgG synthesize (RAG1 and RAG2) and class switching (AID) were also detected in these cells.

In addition, we demonstrated that fertilization could be inhibited when sperm were pre-incubated with antibody to IgG. The sperm appeared to attach to the eggs but did not penetrate zona pellucida-free animal (hamster) ova. This indicates that the IgG molecule on sperm may be essential for fertilization.

Fertilization entails the fusion of an egg with a sperm, which is a multistep process^26^,^27^. Firstly, a mature sperm meet with an egg in the oviduct, driven by special chemoattractant, and then the sperm with an intact acrosome binds to the zona pellucida (ZP) of the egg. After binding to the ZP, sperms have to undergo the acrosome reaction and penetrate the thick extracellular coat to reach and fuse with the egg. Once a single sperm was fused with the egg, the other sperms lost the chance to fuse with the same egg.

The cause of infertility could be multiple and auto-antibody against the sperm is a major cause accounting for 2–30% of infertility^28^,^29^. These anti-sperm antibodies (ASA) produced by male or female can identify the specific antigens on the sperm and impair the function of sperm including motility, capacitation, acrosome reaction and penetration to egg. Besides, ASA can induce the production of cytokines which could influence sperm function, impair sperm-cervical mucus interaction, induce sperm cytotoxicity, increase sperm phagocytosis or inhibit embryo development and implantation^28^. In this study we found that sperm can produce IgG which are mainly located in the neck and the tail regions of sperm. We also found that anti-IgG antibody can inhibit sperm-egg fertilization. This findings suggest that if there was anti-IgG antibody in the environment of fertilization, it could serve as an additional cause for infertility.

An immunoglobulin superfamily protein “Izumo” was reported to be present in the sperms and it was thought to be one of three proteins essential for sperm and egg fusion^27^,^30^. Izumo^—/—^ mutant mice were healthy and showed no obvious abnormalities, but males were sterile. Although the sperm of izumo knockdown mice had normal motility and were capable of binding to eggs to undergo acrosome reaction, they failed to fuse with the egg. In addition, when the Izumo^—/—^ sperm were injected into the cytoplasm of normal eggs, the embryos developed normally. Therefore, the expression of Izumo by sperm appears to be essential in sperm-egg fusion during fertilization.

The molecular mechanism during gamete fusion at fertilization is complicated and has not been fully understood^27^. Following initial contact, the presence of izumo on the sperm and its receptors on the eggs appear to be crucial for penetration and fertilization^31^. In this study, we detected IgG synthesis in sperm and found that anti-IgG antibody could inhibit sperm penetration to egg, suggesting that the sperm derived IgG may be an important factor in sperm-egg fusion. As both IgG and izumo belong to immunoglobulin superfamily with molecular resemblance, the role of each and the possible interactions of the two during fertilization should be further investigated.

In this study, we also detected asymmetric IgG in the sperm. Such antibody was regarded as “blocking antibody” that would play a role of immune protection^19^,^21^,^32^,^33^. It is possible that such asymmetric IgG plays a role of immune protection in fertilization. When there are anti-IgG antibodies produced by B-lymphocytes *in vivo*, the sperm expressed IgG may bind to such IgG and block the subsequent immune effector reactions to sperms.

In summary, we found that IgG can be produced by sperm cells and it might play a role in fertilization and infertility. Understanding the function of sperm produced IgG including aberrantly Fab glycosylated IgG will help to unveil the mechanism that may lead to new therapeutic approaches to sterility.

## Methods

### Ethics Approval of Human Subjects and Animal Experiments

All protocols used in this study involving animals and human subjects were approved by the Ethics Review Committee of Shantou University Medical College according to the guidelines recommended by National Institute of Health involving human subjects and animal care and 1975 Declaration of Helsinki. Informed consent was obtained from all subjects. Human sperm samples of anonymous donors (22–45 years old healthy male, average 27 years old) and mature hamster egg-sperm penetration assay were performed according to established protocols^25^,^34^.

### Preparations of human sperm specimens

Sperm samples (n = 105), which were obtained from undisclosed healthy donators by masturbation after 3 days of abstinence, were kept in a 5% CO_2_ incubator at 37 °C for 30 min to allow liquefaction. The normal semen parameters of sperms were evaluated according to the WHO guidelines^35^. In order to prevent any contamination by other cells, such as premature sperm cells, epithelial cells, sertoli cells or leukocytes, the liquified semen samples were fractionated by the swim-up method as described following: Liquefied (0.2 ml in culture tube) semen sample were lay gently under 2 ml of Bigger-Whittem-Whittingham (BWW) medium containing 0.3% bovine serum albumin (BSA) and then incubated for 1 h at 37 °C in a CO_2_ incubator. The supernatant in each tube was collected and pooled followed by centrifugation at 300 × g for 5 min at room temperature. The pellet of motile sperm was washed once and resuspended in BWW medium with 0.3% BSA to a final concentration of 1 × 10^6^/ml for the subsequent assays.

### Immunofluorescence stain

The prepared motile sperms were incubated at 37 °C in a CO_2_ (5%) incubator for 4 h in a 100 μl drop of capacitated liquid, containing 1 μl of primary antibody such as rabbit anti-human IgG γ chain polyclonal antibody (1:100, Dako, Denmark), rabbit anti-human IgG λchain monoclonal antibody (1:100, ZSGB-BIO, China) or mouse anti-human IgG κ chain polyclonal antibody (1:100, Dako, Denmark) (See detail in Supplementary table S1). These sperm samples were fixed on slides with 4% paraformaldehyde (PFA) for 15 min at room temperature followed by washing with phosphate buffer solution (PBS) and then permeabilized with 0.1% Triton X-100 for 2 min. The Alexa Fluor 594-conjugated (Jackson, Immuno. Research Lab, USA) or Alexa Fluor 488-conjugated (Invitrogen, USA) secondary antibody (Supplementary table S1) was incubated with the sperms for 1h at RT. Nucleus was stained with 4′, 6-diamidino-2-phenylindole (DAPI) (Sigma-Aldrich Co. LLC, USA) and slides were mounted with Vectashield (Vector Laboratories, USA). Sperm samples incubated with PBS without the primary antibodies were used as negative controls. Protein localization was examined at a magnification of ×400 with a fluorescence microscope (Carl Zeiss Inc., USA).

### Immuno-electron microscopy

Human sperm samples were fixed in 2% PFA with 0.2% glutaraldehyde in 0.1M PBS (pH 7.4) at 4 °C overnight. After dehydration in graded ethanol on ice, the samples were penetrated in LR White resin (Ted Pella Inc., USA). Then the samples were polymerized for 5–6 days under ultraviolet light at 4 °C. After that, a glass knife was used to make semi-thin sections (1–2 μm) under a dissection microscope (Motic, China), and the sections were stained with toluidine blue to locate areas of interest. Finally, ultra-thin (70–90 nm) sections, cut with a diamond knife, were collected on 200-mesh nickel grids. To stain the sections, the samples were first blocked in 5% BSA in phosphate buffer solution-tween (PBST) (0.1% Tween 20 in 0.1M PBS) for 1 h, followed by incubation with rabbit anti-human IgG γ chain specific polyclonal antibody (1:100, Dako, Denmark) at 4 °C overnight. After washing, the sections were blocked in PBST for 1 h, and incubated with a secondary antibody for 1 h at room temperature. Then the sections were washed with PBS and distilled water. After the sections were air-dried and stained with 5% uranyl acetate, they were rinsed again in distilled water. Finally, the sections were viewed with a JEOL JEM-1400 transmission electron microscope (TEM) operating at 80 kV.

### Western blot

Cell lysates were prepared from motile sperms using RIPA buffer according to the manufacturer’s instructions (Beyotime, China). Fifty-six micrograms of denatured human sperm total proteins were separated on a 10% SDS-PAGE gel with a Criterion® system (Bio-Rad, USA). Standard human IgG (0.2 μg, 0.5 μg and 0.1 μg respectively, Sigma-Aldrich Co. LLC, USA) was used as a positive control. The separated proteins were then transferred onto a nitrocellulose membrane. After blocked with 5% BSA, the nitrocellulose membrane were incubated with rabbit anti-human IgG γ chain specific polyclonal antibody (1:2500, Dako, Denmark), rabbit anti-human IgG λ chain specific monoclonal antibody (1:250, ZSGB, China), and mouse anti-human IgG κ chain specific polyclonal antibody (1:1000, Doka, Denmark). The appropriate fluorescein-labeled secondary antibodies were used. Finally, the membranes were analyzed in a two-color LI-COR Odyssey Imaging System (LI-COR Biosciences, Lincoln, NE, USA) according to the manufacturer’s protocol.

### RT-PCR assay

Total RNA from motile sperm and Raji cells ((human Burkitt’s lymphoma cell line, positive control, purchased from ATCC (June 12, 2008)) were extracted using a GenEluteTM Mammalian Total RNA Miniprep Kit (Sigma-Aldrich Co. LLC, USA). Reverse transcription (RT) PCR of 5 μg total RNA was performed with the SuperScriptTM III First-Strand Synthesis System for RT-PCR (Invitrogen, USA) according to the manufacturer’s instructions. The RT-PCR reaction was performed at 50 °C for 50 min.

PCR primers used for amplification of cDNA of the constant region of the IGHG1 including those of IgG λ chain and IgG κ chain were as previously described^1^,^4^ and were listed in Supplementary table S2. The sense and antisense primers spanned across introns were applied to facilitate the discrimination between transcripts and genomic DNA. The PCR products were analyzed by separation on 2% agarose gel in electrophoresis.

To analyze IgG V-D-J rearranged gene (RAG1, RAG2) expression, a nested RT-PCR assay was used (Supplementary table S2). Conserved active site of cytidine deaminase was selected as the target with primers as described previously (Supplementary table S2)^1^. For cDNA amplification of RAG1 and RAG2, DNase I (Amplification Grade) (Invitrogen, USA) was used to pretreat RNA samples to exclude any contamination by genomic DNA. The negative control was amplified with DNAase treated RNA as the template. Raji cells were used as the positive controls. Nested RT-PCR was also performed as described previously^36^.

The identification of PCR products was confirmed with DNA sequencing. The PCR primers used for amplification and the expected lengths of the resulting PCR products are presented in Supplementary table S2.

### *In Situ* Hybridization

The cRNA probe against IGHG1 was prepared as described previously^1^. And the probe sequences were validated with DNA sequencing. Briefly, deparaffinized and dehydrated human spleen tissue sections (4 μm) were incubated in 0.1 M HCl for 10 min, and heated to 95 °C in a microwave oven in 0.01 M citrate buffer (pH 6.0) for 20 min. The slides were cooled to room temperature, washed with PBS, fixed in 4% PFA for 10 min, and hybridized overnight at 55 °C with human IGHG1 cRNA probe (sequence: acggcgtggaggtgcataatgccaagacaaagccgcgggaggagca gtacaacagcacgtaccgtg tggtcagcgtcctcaccgtcctgcaccaggactggctgaatggcaaggagtacaagtgcaaggtctccaacaaagccctcccagcccccatcgagaaaaccatctccaaagccaaagggcagccccgagaaccacaggtgtacaccctgcccccatcccgggaggagatgaccaagaaccaggtcagcctgacctgcctggtcaaaggcttctatcccagcgacatcgccgtggagtgggagagcaatgggcagccggagaacaactacaagaccacgcctccc). After hybridization, sections were washed in 2 × SSC plus 50% formamide for 15 min and 2 × SSC twice for 15 min (50 °C) each. The samples were incubated with anti-digoxigenin antibody conjugated with alkaline phosphatase (AP) (dilution 1:500; Roche Diagnostics, Switzerland). 5-Bromo-4-chloro-3-in-dolyl phosphate (BCIP) and nitro-blue-tetrazolium (NBT) (Promega, USA) were used for the color reaction. For controls, slides were incubated with the corresponding sense probes. Finally, all sections were counterstained with methyl green.

### Indirect ELISA to measure the concentration of asymmetric IgG in sperm

It was reported that a certain percentage of IgG in serum and placenta is glycosylated at one of its Fab arms to make the molecule asymmetric^20^. Such asymmetric antibodies are thought to protect instead of attack the molecules they bind to^37^. To detect such IgG, we added 50 μl of 2.6 μg/μl sperm total protein in 1200 μl ConA solution buffer. Then put 1 ml of the dilution into concanavalin A affinity column. Aberrant glycosylated IgG would specifically bind to concanavalin A. We collected unbound solution containing symmetric IgG in 15 ml centrifuge tubes. Then we used 1 ml solution buffer to wash the column, and collected the solution in the 15 ml centrifuge tube again. Human IgG ELISA kit (Immunology Consultants Laboratory, USA) was used to measure the concentration of total IgG and that of symmetric IgG following the manufacturer’s instructions. The percentage of asymmetric IgG was then derived.

### Zona-free hamster egg sperm penetration assay

To test the ability of fertilization of human sperm, the protocols for zona-free hamster egg-sperm penetration assay were performed according to previous report^25^. Briefly, gamete incubations were carried out in micro-drops under paraffin oil at 37 °C in a 5% CO_2_ incubator. The swim-up sperm samples prepared as described were washed twice by centrifugation (5 min at 600 × g) in a 15 ml centrifuge tube. Hamster eggs were obtained from Golden Syrian hamsters by superovulation with PMSG (Pregnant Mare Serum Gonadotropin). Cumulus cells were removed by treating eggs with 0.1% hyaluronidase (Sigma-Aldrich Co. LLC, USA) for 3 min. The eggs were then pooled and washed with BWW medium containing 0.3% BSA with a pulled, heat-polished Pasteur pipette. Zona pellucida (ZP) was removed by treating the eggs with 0.1% trypsin (Sigma-Aldrich Co. LLC, USA) for 30 s followed by washing twice. The eggs were then distributed into the treatment group and the control group. One hour later, the zona-free hamster eggs were examined for penetration by sperms under a phase-contrast microscope after the denuded eggs were compressed between a slide and a coverslip. The zona-free hamster eggs were considered to be fertilized when swollen sperm head or pronucleus was observed inside the eggs with accompanying sperm tails.

### Statistical analysis

Differences in fertilization rates of sperm bound eggs in the negative control groups and the experimental groups were analyzed with one way ANOVA test with Dunnet’s post-hoc test. Values are presented as means ± SE from four independent experiments and differences were considered to be statistically significant at p < 0.05.

## Additional Information

**How to cite this article**: Yan, M. *et al.* Immunoglobulin G Expression in Human Sperm and Possible Functional Significance. *Sci. Rep.* **6**, 20166; doi: 10.1038/srep20166 (2016).

## Supplementary Material

Supplementary Tables

## References

[b1] Chen Z. S. & Gu J. Immunoglobulin G expression in carcinomas and cancer cell lines. Faseb J 21, 2931–2938, 10.1096/fj.07-8073com (2007). 17475920

[b2] Huang J., Zhang L., Ma T., Zhang P. & Qiu X. Expression of Immunoglobulin Gene With Classical V-(D)-J Rearrangement in Mouse Testis and Epididymis. J Histochem Cytochem 57, 339–349, 10.1369/jhc.2008.951434 (2009). 19064717 PMC2664983

[b3] Niu N. *et al.* Expression and distribution of immunoglobulin G and its receptors in the human nervous system. Int J Biochem Cell B 43, 556–563, 10.1016/j.biocel.2010.12.012 (2011). 21167303

[b4] Niu N. *et al.* Expression and distribution of immunoglobulin G and its receptors in an immune privileged site: the eye. Cell Mol Life Sci 68, 2481–2492, 10.1007/s00018-010-0572-7 (2011). 21061041 PMC11114928

[b5] Lei Y. *et al.* Expression and distribution of immunoglobulin G in the normal liver, hepatocarcinoma and postpartial hepatectomy liver. Lab Invest 94, 1283–1295, 10.1038/labinvest.2014.114 (2014). 25264708

[b6] Gu J. *et al.* Fab fragment glycosylated IgG may play a central role in placental immune evasion. Hum Reprod 30, 380–391, 10.1093/humrep/deu323 (2015). 25505012 PMC4303772

[b7] Qiu X. Y. *et al.* Human epithelial cancers secrete immunoglobulin G with unidentified specificity to promote growth and survival of tumor cells. Cancer Res 63, 6488–6495 (2003). 14559841

[b8] Qiu Y. M. *et al.* Immunoglobulin G expression and its colocalization with complement proteins in papillary thyroid cancer. Modern Pathol 25, 36–45 (2012). 10.1038/modpathol.2011.139 21909078

[b9] Allen G. J. & Bourne F. J. Interaction of immunoglobulin fragments with the mammalian sperm acrosome. J Exp Zool 203, 271–276, 10.1002/jez.1402030209 (1978). 342673

[b10] Auer J., Senechal H. & De Almeida M. Sperm-associated and circulating IgA and IgG classes of antibodies recognise different antigens on the human sperm plasma membrane. J Reprod Immuonl 34, 121–136, 10.1016/s0165-0378(97)00023-5 (1997). 9292779

[b11] Hjort T. Quantitative determination of IgG and IgA on sperm from infertile patients with and without antisperm antibodies. Am J Reprod Immunol 36, 211–215 (1996). 8911628 10.1111/j.1600-0897.1996.tb00165.x

[b12] Kimoto Y. Expression of heavy-chain constant region of immunoglobulin and T-cell receptor gene transcripts in human non-hematopoietic tumor cell lines. Gene Chromosome Canc 22, 83–86, 10.1002/(sici)1098-2264(1998)22:1<83::aid-gcc12>3.0.co;2-o(1998 ). 9591639

[b13] Kimoto Y. A single human cell expresses all messenger ribonucleic acids: the arrow of time in a cell. Mol Gen Genet 258, 233–239 (1998). 9645429 10.1007/s004380050727

[b14] Weininger R. B., Fisher S. & Rifkin J. & Bedford, J. M. Experimental studies on the passage of specific IgG to the lumen of the rabbit epididymis. J Reprod Fertil 66, 251–258 (1982). 7120189 10.1530/jrf.0.0660251

[b15] Bronson R. A., Cooper G. W. & Rosenfeld D. L. Correlation between regional specificity of antisperm antibodies to the spermatozoan surface and complement-mediated sperm immobilization. Am J Reprod Immunol 2, 222–224 (1982). 7137446 10.1111/j.1600-0897.1982.tb00170.x

[b16] Knee R. A., Hickey D. K., Beagley K. W. & Jones R. C. Transport of IgG across the blood-luminal barrier of the male reproductive tract of the rat and the effect of estradiol administration on reabsorption of fluid and IgG by the epididymal ducts. Biol Reprod 73, 688–694, 10.1095/biolreprod.105.041079 (2005). 15888731

[b17] Hulse R. E., Swenson W. G., Kunkler P. E., White D. M. & Kraig R. P. Monomeric IgG Is Neuroprotective via Enhancing Microglial Recycling Endocytosis and TNF-alpha. J Neurosci 28, 12199–12211, 10.1523/jneurosci.3856-08.2008 (2008). 19020014 PMC2699401

[b18] Arumugam T. V. *et al.* Intravenous immunoglobulin (IVIG) protects the brain against experimental stroke by preventing complement-mediated neuronal cell death. P Natl Acad Sci USA 104, 14104–14109, 10.1073/pnas.0700506104 (2007). PMC1955802 17715065

[b19] Margni R. A., Perdigon G., Abatangelo C., Gentile T. & Binaghi R. A. Immunobiological behaviour of rabbit precipitating and non-precipitating (co-precipitating) antibodies. Immunology 41, 681–686 (1980). 7461708 PMC1458152

[b20] Labeta M. O., Margni R. A., Leoni J. & Binaghi R. A. Structure of asymmetric non-precipitating antibody: presence of a carbohydrate residue in only one Fab region of the molecule. Immunology 57, 311–317 (1986). 3081439 PMC1453940

[b21] Gentile T., Llambias P., Dokmetjian J. & Margni R. A. Effect of pregnancy and placental factors on the quality of humoral immune response. Immunol Lett 62, 151–157 (1998). 9698113 10.1016/s0165-2478(98)00041-8

[b22] Leoni J., Labeta M. & Margni R. A. The asymmetric IgG non-precipitating antibody. Localization of the oligosaccharide involved, by concanavalin A interaction. Mol Immunol 23, 1397–1400 (1986). 3821742 10.1016/0161-5890(86)90026-x

[b23] Gutierrez G., Gentile T., Miranda S. & Margni R. A. Asymmetric antibodies: a protective arm in pregnancy. Chem Immunol Allergy 89, 158–168, 10.1159/000087964 (2005). 16129962

[b24] Taylor D. D. & Gercel-Taylor C. Tumor-reactive immunoglobulins in ovarian cancer: diagnostic and therapeutic significance? (review). Oncol Rep 5, 1519–1524 (1998). 9769398 10.3892/or.5.6.1519

[b25] Yanagimachi R., Yanagimachi H. & Rogers B. J. The use of zona-free animal ova as a test-system for the assessment of the fertilizing capacity of human spermatozoa. Biol Reprod 15, 471–476, 10.1095/biolreprod15.4.471 (1976). 974200

[b26] Wassarman P. M., Jovine L. & Litscher E. S. A profile of fertilization in mammals. Nat Cell Biol 3, E59–64, 10.1038/35055178 (2001). 11175768

[b27] Klinovska K., Sebkova N. & Dvorakova-Hortova K. Sperm-egg fusion: a molecular enigma of mammalian reproduction. Int J Mol Sci 15, 10652–10668, 10.3390/ijms150610652 (2014). 24933635 PMC4100174

[b28] Restrepo B. & Cardona-Maya W. Antisperm antibodies and fertility association. Actas Urol Esp 37, 571–578, 10.1016/j.acuro.2012.11.003 (2013). 23428233

[b29] Menge A. C., Medley N. E., Mangione C. M. & Dietrich J. W. The incidence and influence of antisperm antibodies in infertile human couples on sperm-cervical mucus interactions and subsequent fertility. Fertil Steril 38, 439–446 (1982). 7117571 10.1016/s0015-0282(16)46578-7

[b30] Inoue N., Ikawa M., Isotani A. & Okabe M. The immunoglobulin superfamily protein Izumo is required for sperm to fuse with eggs. Nature 434, 234–238, 10.1038/nature03362 (2005). 15759005

[b31] Bianchi E., Doe B., Goulding D. & Wright G. J. Juno is the egg Izumo receptor and is essential for mammalian fertilization. Nature 508, 483-+, 10.1038/nature13203 (2014). PMC3998876 24739963

[b32] Zenclussen A. C., Gentile T., Kortebani G., Mazzolli A. & Margni R. Asymmetric antibodies and pregnancy. Am J Reprod Immunol 45, 289–294 (2001). 11432403 10.1111/j.8755-8920.2001.450504.x

[b33] Ercan A. *et al.* Aberrant IgG Galactosylation Precedes Disease Onset, Correlates With Disease Activity, and Is Prevalent in Autoantibodies in Rheumatoid Arthritis. Arthritis and Rheum 62, 2239–2248, 10.1002/art.27533 (2010). 20506563 PMC4118465

[b34] Consensus workshop on advanced diagnostic andrology techniques. ESHRE (European Society of Human Reproduction and Embryology) Andrology Special Interest Group. Hum Reprod 11, 1463–1479 (1996). 8671487

[b35] Organization W. H. WHO laboratory manual for the examination and processing of human semen. J Androl 30, 9–9 (2010). 21243747

[b36] Lynch S., Kelleher D., McManus R. & O’Farrelly C. RAG1 and RAG2 expression in human intestinal epithelium: evidence of extrathymic T cell differentiation. Eur J Immunol 25, 1143–1147, 10.1002/eji.1830250502 (1995). 7774617

[b37] Margni R. A. & Malan Borel I. Paradoxical behavior of asymmetric IgG antibodies. Immunol Rev 163, 77–87, 10.1111/j.1600-065X.1998.tb01189.x (1998). 9700503

