# Supplementary material for: Immunoglobulin G Expression in Human Sperm and Possible Functional Significance
Source: Sci Rep. 2016 Feb 1;6:20166. doi: 10.1038/srep20166 (PMC4735602; doi:10.1038/srep20166)
Supplement: Supplementary Tables [file srep20166-s1.pdf]

Immunoglobulin G Expression in Human Sperm and Possible Functional Significance

Meiling Yan[a†], Xiaoyu Zhang[a†], Qinxue Pu[a†], Tao Huang[a], Qingdong Xie[b], Yan Wang,[c] Jing Li[a], Yun Wang[a], Huan Gu[a], Tianhua Huang[d], Zhiling Li[e] and Jiang Gu[a*]

Supplementary table S1. Antibodies used in this study.

Name	Host species	Clonality	Dilution	Manufacture
Anti-human IgG antibody (γ chain specific)	rabbit	polyclonal	1:100	Dako, Denmark
Anti-human IgG antibody (λ chain specific)	rabbit	monoclonal	1:100	ZSGB-BIO, Beijing, China
Anti-human IgG antibody (κ chain specific)	mouse	polyclonal	1:100	Dako, Denmark
Alexa Fluor® 594 anti-rabbit IgG (H+L)	donkey	polyclonal	1:400	Jackson Immuno. Research Lab, USA
Alexa Fluor® 488 anti-mouse IgG (H+L)	goat	polyclonal	1:400	Invitrogen, USA
Anti-human GAPDH antibody	mouse	polyclonal	1:2000	Santa Cruze, USA

Supplementary table S2. Primers used for PCR amplification.

Gene Name	Primer sequence (5'-3')	
IGHG1	External	ACGGCGTGGAGGTGCATAATG (sense)
		CGGGAGGCGTGGTCTTGTAGTT (anti-sense)
	Internal	GACTGGCTGAATGGCAAGGAG (sense)
		GGCGATGTCGCTGGGATAGAA (anti-sense)
RAG1	External	TGGATCTTTACCTGAAGATG (sense)
		CTTGGCTTTCCAGAGAGTCC (anti-sense)
	Internal	CACAGCGTTTTGCTGAGCTC (sense)
		AGCTTGCCTCAGGGTTCATG (anti-sense)
RAG2	External	TGGAAGCAACATGGGAAATG (sense)
		CATCATCTTCATTATAGGTGTC (anti-sense)
	Internal	TTCTTGGCATACCAGGAGAC (sense)
		CTATTTGCTTCTGCACTG (anti-sense)
AID	External	GAAGAGGCGTGACAGTGCT (sense)
		CGAAATGCGTCTCGTAAGT (anti-sense)
	Internal	CCTTTTCACTGGACTTTGG (sense)
		TGATGGCTATTTGCACCCC (anti-sense)
GAPDH	External	ACCACAGTCCATGCCCATCAC (sense)
		TCCACCACCCTGTTGCTGTA (anti-sense)
	Internal	ACCACAGTCCATGCCCATCAC (sense)
		TCCACCACCCTGTTGCTGTA (anti-sense)
IgG λ	External	GAGCCTGACGCCTGAG (sense)
	Internal	ATTGAGGGTTTATTGAGTGCAG (anti-sense)
IgG κ	External	TGAGCAAAGCAGACTACGAGA (sense)
	Internal	GGGGTGAGGTGAAAGATGAG (anti-sense)
CD19	External	TACTATGGCACTGGCTGCTG (sense)
	Internal	CACGTTCCCGTACTGGTTCT (anti-sense)
